# Position Tracking During Human Walking Using an Integrated Wearable Sensing System

**DOI:** 10.3390/s17122866

**Published:** 2017-12-10

**Authors:** Giulio Zizzo, Lei Ren

**Affiliations:** School of Mechanical, Aerospace and Civil Engineering, University of Manchester, Manchester M13 9PL, UK; giulio.zizzo@manchester.ac.uk or g.zizzo17@imperial.ac.uk

**Keywords:** Kalman filter, pedestrian dead reckoning, wearable sensors, IMU navigation

## Abstract

Progress has been made enabling expensive, high-end inertial measurement units (IMUs) to be used as tracking sensors. However, the cost of these IMUs is prohibitive to their widespread use, and hence the potential of low-cost IMUs is investigated in this study. A wearable low-cost sensing system consisting of IMUs and ultrasound sensors was developed. Core to this system is an extended Kalman filter (EKF), which provides both zero-velocity updates (ZUPTs) and Heuristic Drift Reduction (HDR). The IMU data was combined with ultrasound range measurements to improve accuracy. When a map of the environment was available, a particle filter was used to impose constraints on the possible user motions. The system was therefore composed of three subsystems: IMUs, ultrasound sensors, and a particle filter. A Vicon motion capture system was used to provide ground truth information, enabling validation of the sensing system. Using only the IMU, the system showed loop misclosure errors of 1% with a maximum error of 4–5% during walking. The addition of the ultrasound sensors resulted in a 15% reduction in the total accumulated error. Lastly, the particle filter was capable of providing noticeable corrections, which could keep the tracking error below 2% after the first few steps.

## 1. Introduction

With the recent advances in portable sensing techniques, the use of wearable sensors for pedestrian navigation has been attracting increasing attention. Such systems have numerous applications, such as healthcare monitoring, intelligent environments [1], lower-limb prosthetic tracking, assisted navigation [2], and emergency responder localisation.

Inertial measurement units (IMUs) are ubiquitously used in these systems and can be combined with other sensors such as ultrasound, barometers, and magnetometers. For a portable pedestrian tracking system, sensors need to be small, lightweight, and low-cost. Thus, IMUs based on microelectromechanical system (MEMS) technology are normally used. However, even high-end commercial MEMS IMUs, such as those produced by Xsens [3], suffer from significant drift over time, and various algorithms have been developed to reduce the accumulated errors.

The idea of using zero-velocity updates (ZUPTs) in combination with an extended Kalman filter (EKF) was introduced in [4], although variations of the algorithm were presented in an earlier study [5]. ZUPTs enabled the EKF to make corrections for numerous system parameters. This method was further developed in other works [6] by incorporating zero-angular-rate updates (ZARUs), Heuristic Drift Reduction (HDR), and magnetometers. Closed-loop errors of 2–10% were achieved. Other studies have attempted to improve the stance phase detection to apply ZUPTs more effectively. In [7], a detection algorithm based on gait cycle, combined with a finite-state machine, resulted in a mean position error of 0.89%. In another study [8], gait frequency was extracted, and the optimal thresholds for applying ZUPTs were adaptively calculated. During a challenging 430 m walk with long curved paths and at different walking speeds, this algorithm achieved a 3.5% error compared to a 5.1% error when the thresholds were fixed. The idea of adaptively changing the ZUPT thresholds has been looked at in other works, for example, in combination with a chest-mounted inertial sensor, ZUPT thresholds were more accurately computed in [9], resulting in a ∼2.5% error when running.

The use of multiple IMUs has been investigated. Using two IMU sensors, one on each shoe, enabled the authors in [10] to achieve more accurate tracking. A constraint was imposed in the algorithm by defining that at all times, the two IMUs could not be separated by more than a maximum distance. A ZUPT-aided Kalman filter was employed by incorporating the maximum bound on the foot-to-foot separation. This enabled both feet to be tracked to a higher accuracy, and thus the system had significantly smaller positional errors.

Interestingly, some recent work has been looking at different locations for an IMU, rather then only the foot. In [11], the authors used the Google Glass product as an IMU platform to perform pedestrian tracking. By stabilising the IMU coordinate system and utilising the user’s walking pattern, errors of 2.5% were achieved.

Combined use with other sensors has been a popular route to improving the accuracy of IMU-based systems. Magnetometers have been used in certain studies [6,12]. However, large magnetic fluctuations indoors prevent these from being widely used, although better calibration techniques are being developed [13]. Alternative uses of magnetometers have also been explored. For example, in [14], a fixed magnet was attached to one shoe, and a varying magnetic field during walking was detected. This enabled a more robust detection of the stance phase required to apply ZUPTs, and the study achieved 1.5% loop closure errors.

Additionally, more sophisticated sensors, such as light detection and ranging (LIDAR), have been used. In [15], the user walked in an indoor environment, and a LIDAR sensor was employed to detect nearby walls and provide heading corrections. The integration of this additional sensor proved to be very effective in error reduction. For example, in the case of a long straight line walk, the error was reduced from 11.53% to 1.91% by using a LIDAR sensor.

The detection of motion phases, rather than simply stance and stride, has begun to benefit from the explosion in machine learning-related algorithms [16]. In [17], the authors trained a support vector machine (SMV) to distinguish between six different types of motion. Then, specifically for walking and running, the ZUPTs have their application thresholds adaptively calculated. This achieved promising results with maximum errors of ∼3%. However, the SMV did require a large amount of IMU training data, which needed to be manually collected.

Smartphones have begun to be used as IMU tracking sensors. One advantage of using a smartphone is that one can easily take advantage of pre-existing infrastructure; for example, when using IMUs in combination with iBeacon technology (a recent low-power Bluetooth technology developed by Apple), the authors in [18] achieved an average localisation accuracy of 1.54 m. When using WiFi fingerprinting rather than iBeacon, a mean error of 2.2 m was obtained. In infrastructure-free environments, the advantages of smartphones are much less clear. Indeed, certain studies show that pocket-mounted systems, such as those employing smartphones, can be more robust when it comes to step detection [19]; however, they have yet to achieve the same levels of accuracy as body-mounted sensors [20]. Despite this, by making certain assumptions about the user’s motions and correlating step frequency to step size, some groups have achieved very high levels of accuracy, reaching 98.91% under certain assumptions and limitations of the user’s motion [21].

The use of environmental knowledge to impose boundary constraints on a user’s predicted motion has been investigated [22,23]. By using a particle filter algorithm, submeter tracking accuracy was achieved. However, a significantly increased amount of information needs to be provided as a priori knowledge to the system for the particle filter to be effective.

Many previous foot-mounted systems have employed high-end sensors, for example, Xsens IMUs. Here, we aim to investigate the potential of using cheap IMUs to achieve similar levels of accuracy, and thus we used ADXL345 accelerometers and ITG-3200 gyroscopes, costing ∼£7 each [24,25]. However, for quick prototyping, a pre-assembled breakout board retailing at £30 for both sensors was selected. This significant reduction in cost could potentially open up commercial low-cost pedestrian navigation for everyday use.

The contributions of this study are the following: (1) The potential of a low-cost, equipment-free calibrated IMU for pedestrian navigation is investigated; (2) the improvement in accuracy by using ultrasound sensors for inter-foot ranging measurements is analysed; (3) lastly, the error accumulation across the entire walking process rather than only the loop misclosure, as is normally used in most previous studies, is evaluated. Our system’s core functionality resulted in a hardware bill of £92. We discuss later how this can be reduced by a significant amount.

The paper is structured as follows: Section 2 discusses the hardware used as well as the algorithms employed to perform pedestrian navigation. In Section 3, the results obtained from our system are examined and discussed. Finally, in Section 4, our conclusions are presented and future research directions suggested.

## 2. Materials and Methods

The system is composed of two core components: the IMU sensors, which, via a Kalman filter, track the user, and secondly, an ultrasound sensing system, which takes range measurements between the feet as additional corrections. We first describe the IMU calibration procedures as well as the Kalman filter before moving on to the ultrasound system. Finally, the algorithms employed by the particle filter are discussed.

### 2.1. Calibration

In this work, two identical IMUs mounted adjacent to each other were used. Each IMU contained an ADXL345 accelerometer coupled with an ITG-3200 gyroscope. To accurately calibrate an IMU, the standard approach is to use a mechanical platform that can be oriented in a range of positions and rotated at a precise angular velocity. This device provides accurate reference signals to calibrate accelerometers and gyroscopes. However, for low-cost IMU sensors costing ∼£10, it is often uneconomical or impractical to use such equipment with a cost of many magnitudes more than the sensors. Therefore, a method is required to calibrate the IMUs without the reliance on such equipment.

To solve this problem, several approaches have been proposed for conducting IMU calibration with less requirements of special equipment [26,27,28]. Here, a method that does not rely on any external equipment was used. It was first introduced in [29] for accelerometer calibration and then further developed in [30] to also incorporate gyroscope calibration.

This equipment-free calibration procedure relies on two separate conditions:

1. The magnitude of a static accelerometer’s output must always equal the magnitude of gravity.
2. Should a static, calibrated accelerometer measure the gravity vector to be $G_{1}$ and should it be rotated so that the new gravity vector is $G_{2}$, then $G_{1}$ and $G_{2}$ are related through
  (1) $$G_{2}=RG_{1}$$
  where *R* is a rotation matrix that is calculated from the gyroscope’s angular velocities.

To relate the raw acceleration data, $a_{r}$, to the calibrated value, $a_{c}$, the following equation was applied:

(2) $$a_{c}=E_{a}\left(a_{r}-b_{a}\right)$$

where $E_{a}$ is a diagonally dominant correction matrix representing the scale factors, misalignments, and cross-axis sensitivities, while $b_{a}$ is the accelerometer bias vector.

To obtain the calibration parameters, $E_{a}$ and $b_{a}$, we made use of the first constraint, described above, and therefore minimised the cost function:

(3) $$L_{a}=\sum_{k=1}^{k=N}{\left(||g|\right|}^{2}-\left|\right|a_{c}^{\left\{k\right\}}{\left|\right|}^{2}{)}^{2}$$

where *g* represents the earth’s gravity and *N* is the number of static orientations that the accelerometer is exposed to. The superscript {k} indexes the acceleration vector at the *k*th orientation.

To calibrate the gyroscopes, initially the sensors were held stationary and the output of each axis was taken as the bias, $\omega_{b}$. Then the following equation was used to conduct the calibration:

(4) $$\omega_{c}=E_{\omega }\left(\omega_{r}-\omega_{b}\right)$$

where $E_{\omega }$ is the calibration matrix, $\omega_{r}$ are the raw gyroscope readings and $\omega_{c}$ are the final calibrated results.

To calculate the gyroscope calibration parameters, the following procedure was applied:

1. The IMU containing the gyroscope and accelerometer is held stationary. An initial gravity vector $G_{1}$ is given by the static calibrated accelerometer.
2. The IMU is rotated approximately 180° around a gyroscope axis. Using the second-order integration method as presented by [31], the rotation matrix *R* is obtained. The exact angle by which the IMU is rotated does not matter. The key requirement is that the IMU must be rotated through a large enough angle such that a drift in the calculated angle from the gyroscope is produced.
3. A gravity vector $u_{a}$ at the new position is measured by the accelerometer. On the basis of the second condition defined in Equation (1), this new gravity vector can also be calculated from the rotation matrix *R* and the initial gravity vector $G_{1}$ as $G_{2}=RG_{1}$. In the absence of errors, $u_{a}$ and $G_{2}$ should have the same values.

Thus, the calibration matrix $E_{\omega }$ was obtained by minimising the error between $u_{a}$ and $G_{2}$ through the cost function:

(5) $$L_{\omega }=\sum_{k=1}^{k=N}\left|\right|u_{a}^{\left\{k\right\}}-G_{2}^{\left\{k\right\}}{\left|\right|}^{2}$$

in which *N* is the number of rotations the IMU is exposed to. The superscript {k} indexes the acceleration vectors $u_{a}$ and $G_{2}$ at the *k*th rotation.

This procedure was repeated twice to allow each of the two IMUs to be calibrated individually. From this point on, the two IMU outputs were averaged, enabling them to function as a single, more accurate sensor.

### 2.2. Extended Kalman Filter

Here we used a modified version of the EKF algorithm presented in [6], which can be referred to for the full mathematical description of the EKF. An overview of the EKF used in this work is given below.

One of the key parameters in the algorithm is the error state vector, defined as

(6) δx=[δϕ,δω,δr,δv,δa]

which tracks the errors in the system. All five components in δx are 3 × 1 matrices; δϕ,δr, and δv are the estimated error in orientation, position and velocity, respectively, while δω and δa are the estimated biases for the gyroscope and accelerometer. The EKF therefore provides periodic corrections for the navigation algorithm during motions.

The filter uses two update measurements to correct the calculated position: ZUPTs and HDR.

(1) **Zero Velocity Updates (ZUPTs)**: these assume that when the foot is flat on the floor, its velocity is zero. Therefore, any non-zero velocity resulting from the IMU data is an error.
(2) **Heuristic Drift Reduction (HDR)**: this attempts to limit the drift in yaw by declaring that if the change in yaw, Δψ, between successive footsteps is below a threshold, then it is due to a drift error in the yaw:
  (7) $$m_{\mathrm{HDR}}=\left\{\begin{array}{cc} \Delta \psi , & \mathrm{if}\;\Delta \psi \leq 5^{\circ }\\ 0, & \mathrm{otherwise} \end{array}\right.$$

### 2.3. Step Detection

In order to implement the updates in the EKF, the stance phase of the foot needed to be accurately determined. Here, IMU signals along with the range measures from two position-sensitive detectors (PSDs) were used. The foot was considered to be in stance phase when three conditions were satisfied:

(1) **Proximity Sensing:** The first criteria involves examining the range measurements from the two PSDs. The two sensors are mounted on the toe and heel of the shoe and are aimed downwards. When the system is first initialised, both sensors take range measurements, $D_{int}$, from their mounting position to the ground. If at instant *k*, the PSDs take a range measurement, $D_{k}$, and it is less than or equal to $1.05D_{int}$, then the first condition, $C_{1}$, is fulfilled:
  (8) $$C_{1}=\left\{\begin{array}{cc} 1, & \mathrm{if}\;D_{k}\leq 1.05D_{int}\\ 0, & \mathrm{otherwise} \end{array}\right.$$
(2) **Acceleration:** The second condition relies on the accelerometer readings. If the magnitude of the bias-compensated acceleration, $\left|\right|a_{c}\left|\right|$, falls within the range $9.3\;{\mathrm{ms}}^{-2}\leq \left|\right|a_{c}\left|\right|\leq 10.3\;{\mathrm{ms}}^{-2}$, then the second condition, $C_{2}$, is satisfied:
  (9) $$C_{2}=\left\{\begin{array}{cc} 1, & \mathrm{if}\;9.3\;{\mathrm{ms}}^{-2}\leq \left|\right|a_{c}\left|\right|\leq 10.3\;{\mathrm{ms}}^{-2}\\ 0, & \mathrm{otherwise} \end{array}\right.$$
(3) **Gyroscope:** The final condition is based on the gyroscope signals. If the magnitude of the calibrated gyroscope readings, $\left|\right|\omega_{c}\left|\right|$, is measured to be under 20° s^−1^, then the third condition, $C_{3}$, is met:
  $$C_{3}=\left\{\begin{array}{cc} 1, & \mathrm{if}\;||\omega_{c}\left|\right|<20^{\circ }\;s^{-1}\\ 0, & \mathrm{otherwise} \end{array}\right.$$

### 2.4. Q and R Matrix Tuning

The performance of EKF depends strongly on how the process, *Q*, and measurement noise, *R*, covariance matrices are modelled. For the process noise matrix, *Q*, a method as described in [32] was used. It is assumed that the accelerometer and gyroscope had two dominant sources of error:

1. **Sensor noise:** The gyroscope and accelerometer had standard deviations of 8.1500 × 10^−4^ rad s^−1^ and 0.0381 ms^−2^.
2. **Calibration errors**: After calibration, the accelerometers produced an average error of 0.265% when measuring gravity. It was impossible to obtain a precise estimate of the calibration error for the gyroscopes, as calibrating turntables were not available. Because the gyroscope calibration was based on a less-accurate procedure, an error of 1% of the measured value was used.

These errors were then employed to calculate the process noise matrix, *Q*, on the basis of the procedure in [32]. Although an initial estimate of *Q* was obtained, several factors were not included, such as temperature effects, hysteresis, and *g*-sensitivity.

An approximate modelling process was undertaken for the measurement noise covariance matrix, *R*. It was assumed that the readings were independent of other measurements and that each had a small uncertainty. Hence, *R* was set to a 4 × 4 matrix with diagonal elements of 0.01 and all others set to 0.

### 2.5. Ultrasound

**Figure 1:**
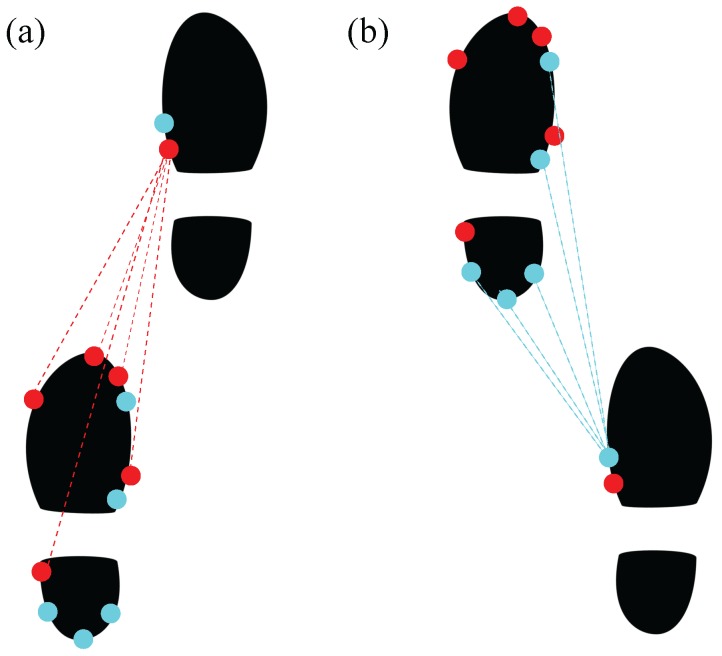
The setup of the ultrasound sensors. The ultrasound sensors marked in red are active when the right leg is the leading leg during walking (**a**). The ultrasound sensors marked in blue are active when the left leg is the leading leg during walking (**b**).

An ultrasound system capable of measuring step lengths during walking was developed. We used HC-SR04 ultrasound sensors in our system. The receivers of the ultrasound system were mounted on the left foot along with the IMUs. Five ultrasound receivers pointing forwards were placed in the front part of the foot, whereas another five receivers pointing backwards were placed in the rear part. On the right foot, two ultrasound transmitters were mounted with one pointing forwards and one pointing backwards (see Figure 1). 

When both feet are on the ground, the relative foot positions can be estimated using the ultrasound sensors. In this scenario, the foot location, O(x,y), is considered to be the intersection of all the circles centered at the position of each ultrasound receiver, $B(x_{i},y_{i})$, which is defined as

(10) $${\left(x-x_{i}\right)}^{2}+{\left(y-y_{i}\right)}^{2}=r_{i}^{2}$$

where $r_{i}$ is the range measurement form the *i*th receiver.

The above equation can be re-expressed by the following linear system:

(11) $$A\hat{x}=b$$

where

(12) $$A=\left[\begin{array}{cc} x_{2}-x_{1} & y_{2}-y_{1}\\ x_{3}-x_{1} & x_{3}-x_{1}\\ \vdots & \vdots \\ x_{n}-x_{1} & x_{n}-x_{1} \end{array}\right]$$

(13) $$\hat{x}=\left[\begin{array}{c} x-x_{1}\\ y-y_{1} \end{array}\right]$$

(14) $$b=\frac{1}{2}\left[\begin{array}{c} r_{1}^{2}-r_{2}^{2}+{\left(x_{2}-x_{1}\right)}^{2}+{\left(y_{2}-y_{1}\right)}^{2}\\ r_{1}^{2}-r_{3}^{2}+{\left(x_{3}-x_{1}\right)}^{2}+{\left(y_{3}-y_{1}\right)}^{2}\\ \vdots \\ r_{1}^{2}-r_{n}^{2}+{\left(x_{n}-x_{1}\right)}^{2}+{\left(y_{n}-y_{1}\right)}^{2} \end{array}\right]$$

Without noise, only three beacons are needed to obtain O(x,y). However, when errors arise in both the range measurements and the positions of $B(x_{i},y_{i})$, then any solution calculated through the above would be inaccurate. Hence, a more sophisticated treatment is required.

Defining the vector *R* to be

(15) $$R=\left[\begin{array}{c} x\\ y \end{array}\right]$$

then the least-squares method gives a good initial estimate of the foot location, $R_{1}$:

(16) $$R_{1}={\left(A^{T}A\right)}^{-1}A^{T}b$$

To improve the results further, a non-linear least-squares algorithm as presented in [33,34] was used. Defining $f_{i}$ to be

(17) $$f_{i}\left(x,y\right)=\sqrt{{\left(x-x_{i}\right)}^{2}+{\left(y-y_{i}\right)}^{2}}-r_{i}$$

where $r_{i}$ is the range measurement of the *i*th beacon, then the Newton iteration gives

(18) $$R_{k+1}=R_{k}-{\left(J_{k}^{T}J_{k}\right)}^{-1}J_{k}^{T}{\hat{f}}_{k}$$

in which

(19) $$J=\left[\begin{array}{cc} \frac{\partial f_{1}}{\partial x} & \frac{\partial f_{1}}{\partial y}\\ \frac{\partial f_{2}}{\partial x} & \frac{\partial f_{2}}{\partial y}\\ \vdots & \vdots \\ \frac{\partial f_{n}}{\partial x} & \frac{\partial f_{n}}{\partial y} \end{array}\right],\hat{f}=\left[\begin{array}{c} f_{1}\\ f_{2}\\ \vdots \\ f_{n} \end{array}\right]$$

We used Equation (16) to give an initial value for *R* and then iterated Equation (18) until convergence was achieved.

The step displacement as measured by the ultrasound sensors was then combined with the IMU-derived step displacement using a weighted average. Therefore, the uncertainties of the two estimated results needed to be determined. Here, we assumed that the error in the navigation frame for each sensor was proportional to the *x* and *y* displacements measured by the individual sensors. This was motivated by the experimental observation that the larger the step size, then the larger the positional error became. This arose predominantly from yaw drift errors in the IMU case and the baseline-to-range ratio for the ultrasound sensors. If the ultrasound data was not available over a footstep, for example, when the line of sight between the receiver and the transmitter was blocked, the system navigation solely relied on the IMUs.

**Figure 2:**
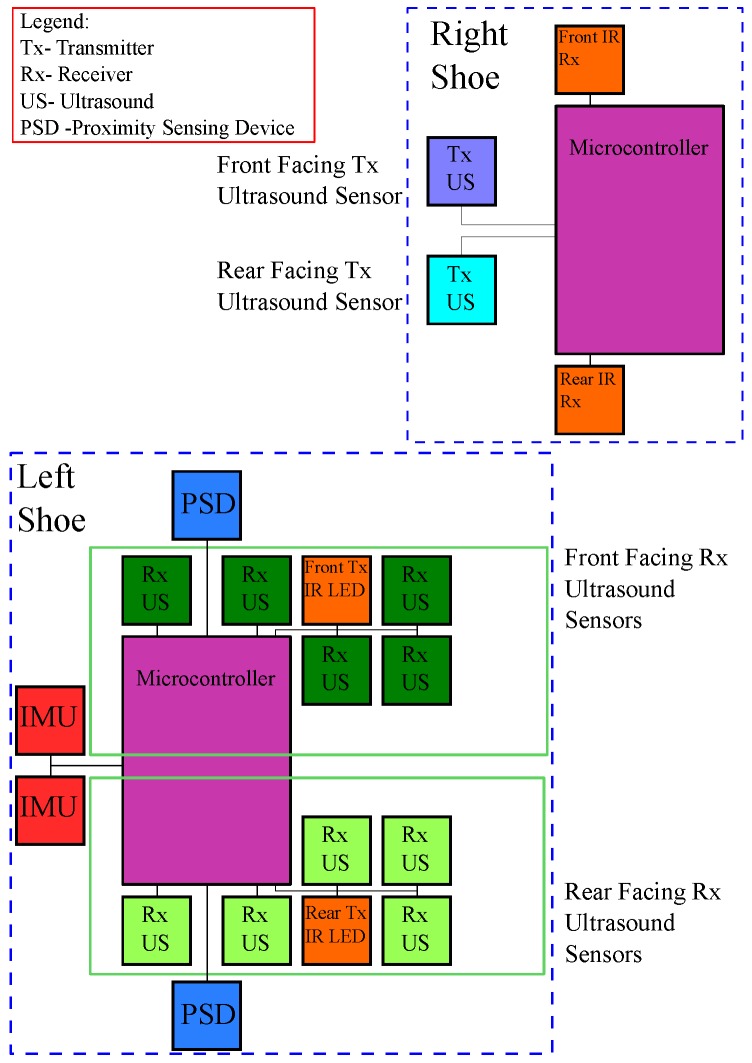
Block diagram of the inertial measurement unit and ultrasound sensors (IMU/US) system setup. The infrared (IR) LEDs are used to synchronise the ultrasound transmitters and ultrasound receivers. The sensors are connected to an ATMega328P micro-controller, which relays the information to a desktop PC for data-processing.

This system consisting of IMUs with integrated ultrasound corrections is referred to as the IMU/US system. The hardware components are illustrated in Figure 2 to give a graphical illustration of how the various components described interface with each other.

### 2.6. Particle Filtering

Unconstrained navigation using the IMU/US system suffers from the gradual accumulation of errors. To improve the performance, an optional particle filtering algorithm was implemented.

The particle filter algorithm receives the step parameters from the underlying IMU/US system and propagates the particles accordingly. Should the particles cross a wall, they are assigned a weight of $1/10000\;N_{p}$, while if no walls are intersected, they are assigned a weighting of $1/N_{p}$, where $N_{p}$ is the number of particles.

**Figure 3:**
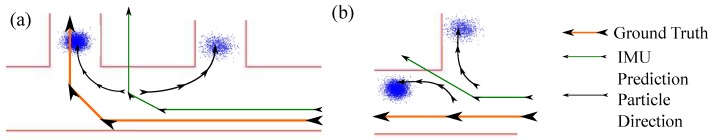
Both (**a**) and (**b**) demonstrate how the particle cloud can diverge. This clearly shows how computing a direct average will yield inaccurate results, as in this case it will give a location situated in an impassible terrain feature. By applying a clustering algorithm, it is possible to exclude the smaller particle cloud from influencing the calculated position.

After resampling the particles, the user’s location needs to be determined. To do so, a subtractive clustering scheme was implemented. The reason that a clustering scheme was used rather than simply an average position was to avoid the effects that could arise if the particle cloud splits. In symmetric environments, a group of particles may diverge from the main particle body. In this scenario, taking an average of all the particles would yield poor results because a large number of particles may end up in incorrect positions, and furthermore, the average location of all the particles may place the user in a physically impossible position. We consider the two scenarios presented in Figure 3:

As we can see, taking an average places the user in a physically impossible position. Therefore, the subtractive clustering scheme as proposed in [35] was used. The underlying principle is to assign a potential to every particle on the basis of the numbers and distances of the other particles. The particle with the highest potential is taken as the cluster centre. Usually, the subtractive clustering algorithm would reduce the potentials of particles near the first cluster centre and then proceed to find more cluster centres. However, this is unnecessary in our case, as the user is only in one position. Thus, the first cluster centre is taken as the true location of the user.

We call this implementation the IUP system, referring to the inertial/ultrasound/particle filter algorithms.

### 2.7. Cost and Form Factor

Currently, the whole sensing system is installed on a preliminary three-dimensional (3D) printed case, which may not be socially acceptable for daily uses. A slimmer and more aesthetically pleasing housing needs to be developed in order for the system to become a viable wearable piece of technology. However, the majority of the current housing is due to the ultrasound system, whereas the IMU system only uses a minimal amount of space and can be used separately in daily life. One of the aims of this study is to investigate how low-cost sensing systems function in place of high-end sensors. The core functionality (Kalman filter-aided inertial navigation) incurred a bill of just over £90. Should a user wish to include ultrasound modules, this incurred us a cost of £50.

Although this places the total price higher than would be realistically affordable for this type of functionality, we make the following observations. Firstly, this is several factors less than an Xsens IMU that would give similar performance. Secondly, and more importantly, this study has demonstrated the performance achievable from this level of hardware quality. We did not conduct an investigation into the production and manufacturing of how a commercial system such as this would be conducted. This would result in a very comfortable reduction in price. The authors believe the £60 total price is entirely achievable. As an illustration, our Arduino boards with ATMega328 micro-controllers are ∼£20 each, depending on the supplier. However, they contain a huge amount of unneeded functionality. Working directly with the ATMega328 micro-controllers (∼£2) and placing the components on printed circuit boards (PCBs) designed in house would already bring us closer to the type of system that would exist in production. Pre-existing off-the-shelf products were selected for the speed and flexibility offered when conducting initial research rather than for their being indicative of the final hardware configuration.

## 3. Results

The performance of the various systems were evaluated for two different types of walking:

1. The first type of walk was carried out in a gait laboratory, and the subject was instructed to walk three times round a rectangular area of approximately 4 m × 2 m. A Vicon motion capture system was used to obtain the ground truth data. The results produced by the wearable sensing system was validated against the Vicon measurement data. This is referred to as Type 1 walking.
2. The second type of walk was conducted in a typical indoor environment. The total walk length measured approximately 55 m, whereby the subject entered and exited several rooms connected by a corridor. In this situation, a Vicon motion capture system was unavailable, and hence the performance of the wearable sensing system was assessed in terms of the final loop misclosure. This type of walk is referred to as Type 2 walking.

For Type 1 walking, two different errors were calculated:

1. **Absolute Error**: This was calculated by measuring the difference between the ground truth determined by the Vicon system and the positions provided by the wearable sensing system. The absolute error was calculated at every single footstep.
2. **Percentage Error**: This was calculated by expressing the absolute error as a percentage of the total distance travelled up to a specific step according to the Vicon system.

The sensor data was streamed to a laptop PC and post-processed. It should be noted that the navigation and EKF algorithms were executed at a frequency slightly below 3000 Hz on the tested PC. Therefore, with suitable software modifications of the data acquisition methods, the algorithms can easily be run in real time. The particle filter algorithm is however, in its current implementation, too slow to run in real time. It would require either a more effective implementation or a more sophisticated algorithm, such as dynamically adapting the number of particles [22], to run in a real-time manner.

### 3.1. Type 1 Walking

**Figure 4:**
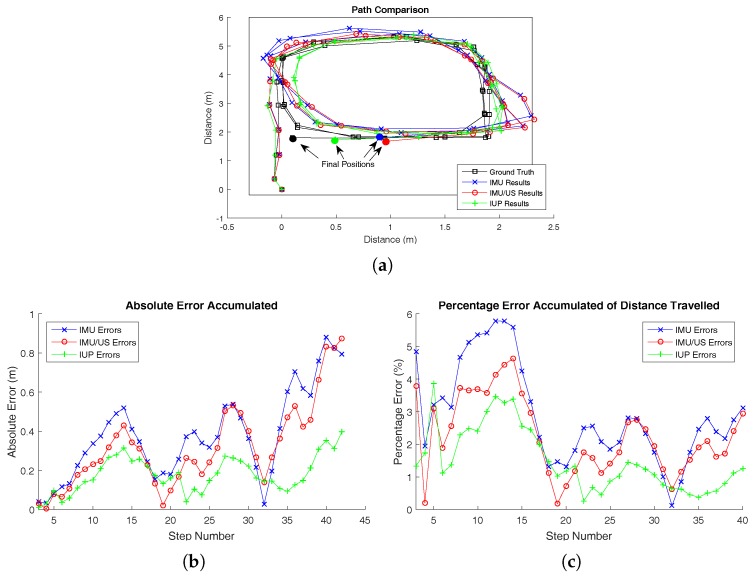
(**a**) The tracked walking paths by the inertial measurement unit (IMU), IMU/ultrasound (US) and inertial/ultrasound/particle filter (IUP) systems for trial 1 of Type 1 walking compared to the ground truth position measured simultaneously by a Vicon motion capture system. (**b**) The absolute errors of the IMU, IMU/US and IUP systems at each single step for trial 1 of Type 1 walking. (**c**) The percentage errors of the IMU, IMU/US and IUP systems at each single step for trial 1 of Type 1 walking.

**Figure 5:**
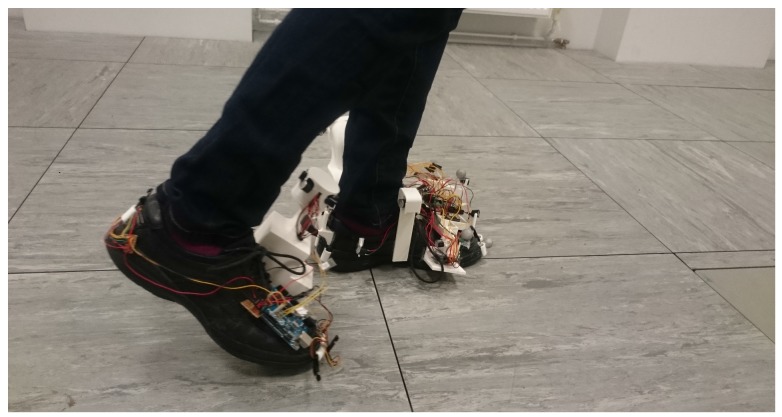
The wearable sensing system during Type 1 walking. The left foot is on the ground when the zero-velocity updates (ZUPT) and Heuristic Drift Reduction (HDR) corrections are applied.

Three Type 1 walking trials were conducted. The results of the first trial are shown here, while the results of the other two trials are presented in Appendix A.1. The walking path for trial 1 is illustrated in Figure 4 along with the associated errors, both in absolute and percentage terms. The system, while being worn and in use, is shown in Figure 5.

For Type 1 walking, the first footstep was used to align the IMU sensor frame with the Vicon coordinate system. In the subsequent steps, the errors between the two systems were analysed.

It can be seen from the figures that walking in a closed loop results in a significant error reduction. In the case of the first walking trial, this resulted in a cyclic error pattern repeating around every 17 steps in Figure 4. Systematic errors are cancelled out in closed-loop walking, and therefore this type of walking pattern results in a much better system performance. Several previous studies have evaluated the system performance only in closed-loop environments; therefore the reported accuracies may have been somewhat overestimated. As it is shown in Figure 4, this is an unrepresentative metric for the average accuracy a user would typically experience.

Figure 4 further shows that the path calculated by the sensing system tends to drift to the left of the ground truth. This could be due to several potential sources. For example, if the IMU slips during every stance phase, a systematic position error may occur. However, this error is almost eliminated over a complete closed-loop path. This error cancellation was significant, as the percentage error oscillated between 4% and 0.5% for all of the three trials.

The figures show that the incorporation of the ultrasound system (IMU/US) resulted in a ∼15% reduction in the total error. However, the ultrasound sensors do not provide any information to correct the drift in yaw, which remains a dominant source of error.

The effect of the particle filter is clearly demonstrated. Incorporating the known map into the particle filter, it is able to keep the maximum accumulated error under 0.3 m in the majority of footsteps. We can also see that the particle filter largely eliminates the cyclic error pattern.

To gain a more quantitative description of how the ultrasound and particle filter systems reduced the error, the areas under the absolute and percentage error graphs (seen in Figure 4b,c for trial 1) were calculated and are shown in Table 1.

### 3.2. Type 2 Walking

**Figure 6:**
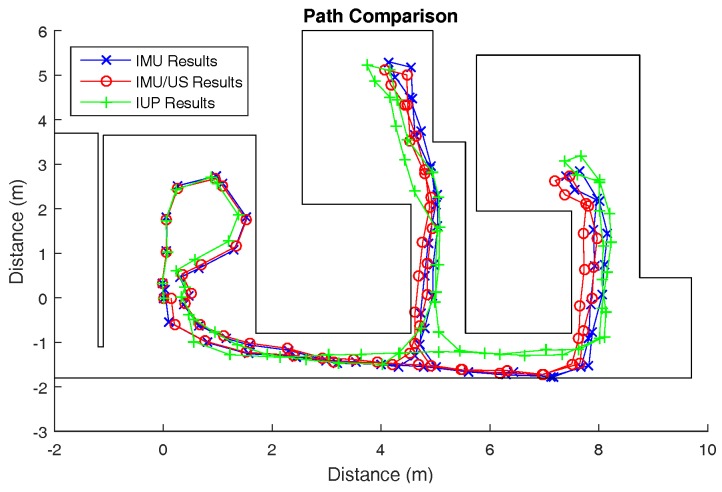
The tracked walking paths by the inertial measurement unit (IMU), IMU/ultrasound (US) and inertial/ultrasound/particle filter (IUP) systems for trial 1 of Type 2 walking.

Three Type 2 walking trials were conducted. Similarly to Type 1 walking, the results of the first trial are shown here, while the results of the other two trials are presented in Appendix A.2. The walking path for trial 1 is illustrated in Figure 6.

In Type 2 walking, the sensing system was examined in a more “natural” scenario. The measurement involved travelling inside a typical indoor environment with the user entering and exiting multiple rooms connected by a corridor. A Vicon motion capture system was not installed in the rooms. Thus, we used the conventional method to evaluate the system performance on the basis of loop misclosure (shown in Table 2). However, as pointed out in the preceding sections, loop misclosure is a vague indication of system performance. As a result of error cancellation in closed-loop paths, the misclosure errors measured were consistently low.

To obtain a better measure of the system accuracy, an approximate maximum error was calculated for the IMU and IMU/US systems. The maximum error was a rough estimate, as accurate ground truth data was not available. To derive the estimate, markers were placed on the floor with known locations that corresponded to key landmark points in the user’s walk, for example, room entry points and U-turns. Then from our results, the maximum difference between the prediction of all the landmark points and their ground truth location was computed. We emphasise that this is a crude measure and it included as a reinforcement to the idea that when walking in a closed-loop fashion, the maximum error is never at the loop misclosure, but always occurs part way through the walk because of systematic error cancellation. This is shown in Table 3, where we can see that the final loop misclosure is always significantly lower than the estimated maximum error. The estimated maximum error was not calculated for the IUP system, as the particle filter’s accuracy along the entire path was high. Therefore to draw meaningful maximum error calculations, a more accurate ground truth measure would have been required for the IUP case.

For Type 2 walking, it was not possible to obtain an accurate measure of the initial heading offset between the IMU and the global frame, as accurate ground truth data was not available. However, the initial heading offset did not influence the loop misclosure calculation. Moreover, the initial heading offset only had a limited effect on the estimated maximum error compared to other error sources.

In the case of Type 2 walking, the IMU/US system performed essentially equivalently to the IMU system. This was due to the fact that the Type 2 walk was more “curvy”. In curving sections, the ultrasound system often does not have an adequate line of sight between the receivers and transmitters, and thus tracking is conducted solely with the IMU. This leads to the ultrasound system being used in a more asymmetric fashion. Therefore, if the walk contains a mixture of curving and straight lines, systematic errors due to the ultrasound are not always cancelled out in closed-loop walking. For example, if the user walks in a straight line forward, then systematic errors from the ultrasound accumulate. However, when the user returns, if they do so in a curvy, looping walk, then the ultrasound system will not function well in the curvy, looping section. Therefore, systematic error cancellation will not occur.

## 4. Conclusions

In this study, a low-cost wearable sensing system, which is capable of tracking the wearer’s position during walking, has been developed and implemented. The system employs an inertial-based navigation algorithm, which received corrections from an EKF. In parallel, an optional ultrasound trilateration system can be used to provide more accurate step-length data. Moreover, a particle filter based on map information can also be incorporated, providing strong error corrections.

The performance of the presented sensing system was compared to the other systems based on higher-end IMUs. To be consistent with the assessments conducted in previous studies, the loop misclosures as given by Type 2 walking unsing only IMU data are used for comparison. Our sensing system incurred loop-closure errors of 0.32%, 0.58% and 1.01% respectively, which were comparable to the performances of the systems developed by previous studies. Errors of 2–10% were obtained when using ZUPTs and HDR in [6]. A worst drift case of 1.28% was found in [36], and a 0.3% error was achieved in [4]. This shows that the low-cost sensing system presented here has a comparable accuracy to the other systems based on high-end IMUs.

When combining IMU sensors with ultrasound, we saw a reduction of ∼15% in the total accumulated error. This result was obtained by comparing the results of our system with those of a Vicon motion capture system as ground truth.

This study shows that the particle filtering algorithm is capable of maintaining yaw accuracy by using map information. This prevents the drift errors from increasing in an unbounded fashion. Indeed, the use of map information resulted in a bounded error, which was below 2% after the first few steps.

From the Type 1 walking results, we found that the misclosure errors of closed-loop walking were significantly reduced as a result of systemic error cancellation. This suggests that previous studies may have underestimated the system errors when using misclosure to assess accuracies. Over a typical closed-loop walk, the errors would oscillate between a maximum of 4% and then would gradually reduce to well under 1% when the user returned to the origin.

Further work may involve the use of magnetometers to remove the yaw drift and thereby obtain a more accurate heading. A more comprehensive validation of the wearable sensing system could be conducted, for example, by using longer walks or walking in more complex environments.

## Figures and Tables

**Table 1:** The cumulative absolute and percentage errors of inertial measurement unit (IMU), IMU/ultrasound (US) and inertial/ultrasound/particle filter (IUP) systems for all three trials during Type 1 walking. For trial 1, this corresponds to the area under the curves in Figure 4b,c. As these are cumulative errors across all the steps taken, they are not true percentages or distances, and hence the cumulative percentage error is not bound in the range 0–100%.

Trial	Cumulative Absolute Error (m)			Cumulative Percentage Error		
	IMU	IMU/US	IUP	IMU	IMU/US	IUP
1	14.80	12.42	6.956	108.5	84.35	56.65
2	19.84	16.45	7.323	140.6	104.2	52.05
3	14.65	13.15	6.068	100.7	97.53	53.90

**Table 2:** The loop misclosure and percentage errors of inertial measurement unit (IMU), IMU/ultrasound (US) and inertial/ultrasound/particle filter (IUP) systems for all three trials during Type 2 walking.

Trial	Loop Misclosure (m)			Percentage Error		
	IMU	IMU/US	IUP	IMU	IMU/US	IUP
1	0.176	0.143	0.592	0.319	0.26	1.07
2	0.319	0.551	0.423	0.579	1.00	0.77
3	0.553	0.439	0.326	1.005	0.79	0.59

**Table 3:** The loop misclosure and estimated maximum errors of inertial measurement unit (IMU) and IMU/ultrasound (US) systems for all three trials during Type 2 walking.

Trial	Loop Misclosure (m)		Estimated Maximum Error (m)	
	IMU	IMU/US	IMU	IMU/US
1	0.176	0.143	0.47	0.48
2	0.319	0.551	1.19	0.92
3	0.553	0.439	1.80	1.74

**Figure A1:**
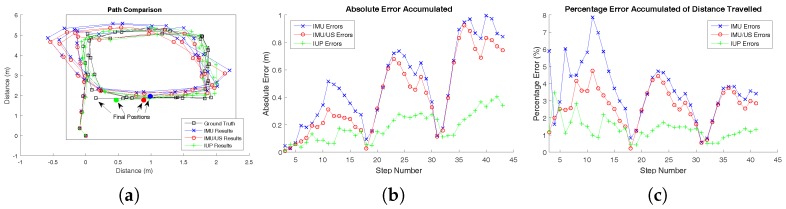
(**a**) The tracked walking paths by the inertial measurement unit (IMU), IMU/ultrasound (US) and inertial/ultrasound/particle filter (IUP) systems for trial 2 of Type 1 walking compared to the ground truth position measured simultaneously by a Vicon motion capture system. (**b**) The absolute errors of the IMU, IMU/US and IUP systems at each single step for trial 2 of Type 1 walking. (**c**) The percentage errors of the IMU, IMU/US and IUP systems at each single step for trial 2 of Type 1 walking.

**Figure A2:**
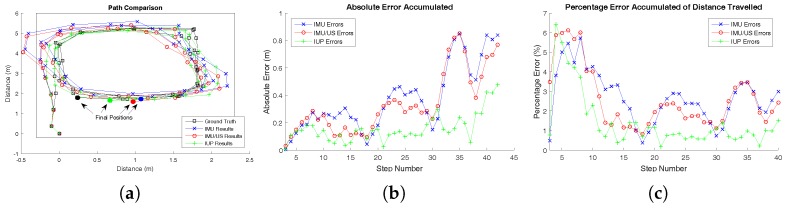
(**a**) The tracked walking paths by the inertial measurement unit (IMU), IMU/ultrasound (US) and inertial/ultrasound/particle filter (IUP) systems for trial 3 of Type 1 walking compared to the ground truth position measured simultaneously by a Vicon motion capture system. (**b**) The absolute errors of the IMU, IMU/US and IUP systems at each single step for trial 3 of Type 1 walking. (**c**) The percentage errors of the IMU, IMU/US and IUP systems at each step for trial 3 of Type 1 walking. In this trial, the percentage error was initially high. This was probably due to the error accumulation in the first step. However, we can see a drop off in the percentage error as the periodic walking pattern began.

**Figure A3:**
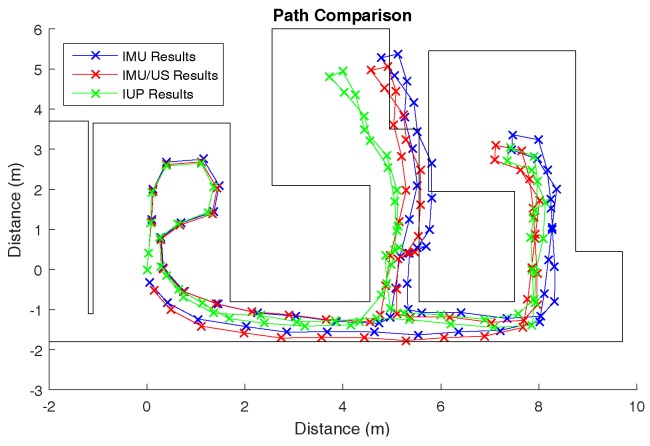
The tracked walking paths by the inertial measurement unit (IMU), IMU/ultrasound (US) and inertial/ultrasound/particle filter (IUP) systems for trial 2 of Type 2 walking.

**Figure A4:**
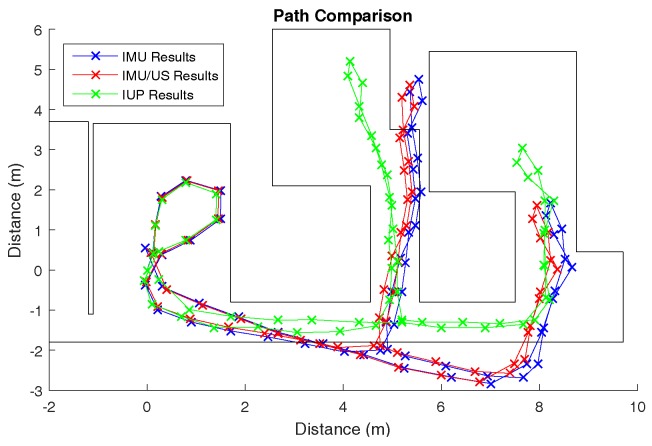
The tracked walking paths by the inertial measurement unit (IMU), IMU/ultrasound (US) and inertial/ultrasound/particle filter (IUP) systems for trial 3 of Type 2 walking.

## Appendix A.

### Appendix A.1. Type 1 Walking Results

The remaining two trials for Type 1 walking are shown here.

### Appendix A.2. Type 2 Walking Results

The remaining two trials for Type 2 walking are shown here.
